# Thorax, Trachea, and Lung Ultrasonography in Emergency and Critical Care Medicine: Assessment of an Objective Structured Training Concept

**DOI:** 10.1155/2013/312758

**Published:** 2013-11-27

**Authors:** Raoul Breitkreutz, Martina Dutiné, Patrick Scheiermann, Dorothea Hempel, Sandy Kujumdshiev, Hanns Ackermann, Florian Hartmut Seeger, Armin Seibel, Felix Walcher, Tim Oliver Hirche

**Affiliations:** ^1^Frankfurter Institut für Notfallmedizin und Simulationstraining, Fachbereich Medizin der Johann Wolfgang Goethe-Universität, Klinikum der Johann Wolfgang Goethe-Universität, 60528 Frankfurt am Main, Germany; ^2^Zentrale Notaufnahme, Klinikum Frankfurt Höchst, Gotenstaße 6–8, 65929 Frankfurt am Main, Germany; ^3^Klinik für Anaesthesiologie, Operative Intensivmedizin und Schmerztherapie, Klinikum Hanau, 63450 Hanau, Germany; ^4^Klinik für Anaesthesiologie, Klinikum der Universität München, Campus Großhadern, 81377 München, Germany; ^5^II. Medizinische Klinik und Poliklinik, Universitätsmedizin Mainz, 55131 Mainz, Germany; ^6^Abteilung Pneumologie/Allergologie, Klinikum der Johann Wolfgang Goethe-Universität, 60528 Frankfurt am Main, Germany; ^7^Institut für Biostatistik und Mathematische Modellierung, Zentrum der Gesundheitswissenschaften, Klinikum der Johann Wolfgang Goethe-Universität, 60528 Frankfurt am Main, Germany; ^8^Abteilung Kardiologie, Klinikum der Johann Wolfgang Goethe-Universität, 60528 Frankfurt am Main, Germany; ^9^Abteilung Anästhesiologie, Intensiv- und Notfallmedizin, Diakonie Klinikum Siegen, 57074 Siegen, Germany; ^10^Klinik für Unfall-, Hand- und Wiederherstellungschirurgie, Klinikum der Johann Wolfgang Goethe-Universität, 60528 Frankfurt am Main, Germany

## Abstract

*Background and Study objective*. Focused lung ultrasound (LUS) examinations are important tools in critical care medicine. There is evidence that LUS can be used for the detection of acute thoracic lesions. However, no validated training method is available. The goal of this study was to develop and assess an objective structured clinical examination (OSCE) curriculum for focused thorax, trachea, and lung ultrasound in emergency and critical care medicine (THOLUUSE). *Methods*. 39 trainees underwent a one-day training course in a prospective educational study, including lectures in sonoanatomy and -pathology of the thorax, case presentations, and hands-on training. Trainees' pre- and posttest performances were assessed by multiple choice questionnaires, visual perception tests by interpretation video clips, practical performance of LUS, and identification of specific ultrasound findings. *Results*. Trainees postcourse scores of correct MCQ answers increased from 56 ± 4% to 82 ± 2% (mean± SD; *P* < 0.001); visual perception skills increased from 54 ± 5% to 78 ± 3% (*P* < 0.001); practical ultrasound skills improved, and correct LUS was performed in 94%. Subgroup analysis revealed that learning success was independent from the trainees' previous ultrasound experience. *Conclusions*. THOLUUSE significantly improves theoretical and practical skills for the diagnosis of acute thoracic lesions. We propose to implement THOLUUSE in emergency medicine training.

## 1. Introduction

Focused lung ultrasound (LUS) examinations are increasingly important diagnostic tools in emergency and critical care medicine [1–4]. A broad area of medicine, including internal medicine [5, 6] and traumatology [1, 7], can benefit from its time and cost-effectiveness and absence of radiation exposure, as well as the reduction of need for transportations of ventilated patients. Using portable ultrasound devices, LUS is now available in virtually all in- and out-of-hospital scenarios [8].

However, unlike focused assessment with sonography in blunt trauma (FAST) that has become a standard procedure, ultrasound examination of thorax and lungs has only recently been established for workup of critically ill patients [9]. Still, when a lesion is suspected, chest X-rays (CXR) or CT scans remain the diagnostic procedures of choice. Apart from echocardiography, noninvasive examination of the chest by ultrasound is not routinely performed in most centers although many investigations support its use [4, 10, 11].

While the detection of fluid such as in hematothorax or pleural effusion (PLE) has been described as relatively simple [12] and ultrasound-guided thoracocentesis can be safely performed in ventilated patients [5], sonographic diagnosis of pneumothorax (PTX) represents a challenge to most examiners. Using a panel of previously validated ultrasound findings and artifacts, for example, absence of lung sliding and comet tails [13, 14] or presence of lung point phenomenon in the M-mode scan, it was demonstrated that diagnosis of PTX could be achieved in real time and with high sensitivity and specificity [4, 15, 15, 16]. There is evidence that LUS allows reliable detection of PTX under conditions (e.g., prehospital) where CXR or CT is not available [1].

To our knowledge, no focused training method for chest sonography, particularly for the use in emergency situations, has been validated until today.

Therefore, we designed an objective structured clinical examination (OSCE) [17] training concept for focused thorax, trachea, and lung ultrasound in emergency and critical care medicine (THOLUUSE). In the present study, we aimed to test if our training concept can improve (1) factual knowledge of theoretical background, (2) visual perceptive skills, and (3) practical imaging performance, that is, the ability to steer the ultrasound probe [18], obtain reproducible image scans, and interpret structures or artifacts during ultrasound examination of the chest.

## 2. Materials and Methods

### 2.1. Study Design

We performed a prospective educational study with a standardized OSCE curriculum. A total of 54 trainees were enrolled into the program in 4 independent training days. Group A included fourteen medical students (age 25 ± 2 years (mean ± standard deviation)) and group B thirty-two anesthesiologists (32 ± 5). Both groups had none or very limited ultrasound knowledge. Group C consisted of eight trauma surgeons (37 ± 6), who all previously underwent a FAST training [19]. Instructors were ultrasound-trained pulmonologists (*n* = 3), internists (*n* = 3), cardiologists (*n* = 4), anesthesiologists (*n* = 2), and trauma surgeons (*n* = 2). Medical interns (*n* = 3) were specifically trained to give instructions for use of ultrasound phantoms. A formal institutional review board approval was obtained at the University of Frankfurt, Medical faculty. All trainees gave informed consent for anonymous analysis of their test results. Patients or relatives of ventilated patients gave their informed consent to serve for teaching purposes according to local ethical standards at our institution.

### 2.2. Course Curriculum and Training System

Theoretical training was scheduled for 2.5 hours and included six brief lectures of anatomy, physiology, and pathology of the thorax, as well as four case presentations on clinical scenarios related to PLE and PTX (Table 1). Practical training was scheduled for 2.5 hours and included two units of hands-on training (HOT), following modifications of previously described OSCE protocols [20, 21]. In HOT-1, each trainee had to pass six and in HOT-2 seven different thematic stations (Table 2). There was a strictly organized circuit system between the HOT stations. The ratio of instructor per station and trainee was 1 : 1 and 1 : 2 offering each trainee at least 5 min of training per station. Each instructor gave a short introduction to the specific objective of the station, followed by a one-minute demonstration of the standardized sonographic views or procedures. In total, twelve major sonographic views or artifacts were studied in healthy models. The same healthy models were used in HOT-1 and -2, but views and structures alternated between individuals. Two stations in HOT-2 included patients with selected pathologies (Table 2). Patients with chronic or malignant lung disease were included to demonstrate PLE; patients that recently underwent sterno- or thoracotomy due to cardiac or pulmonary disease were used to demonstrate PTX. Both HOT-1 and HOT-2 included one virtual station, where typical pathologic findings and artifacts were demonstrated in the form of electronic pictures and video clips on a laptop computer (Table 3).

### 2.3. Phantoms and Ultrasound Equipment

**Figure 1:**
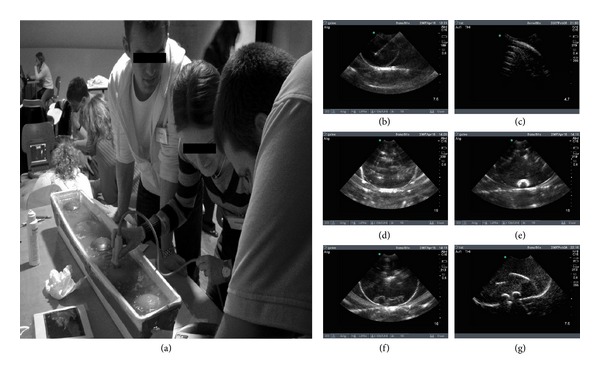
(a) Representative training station with ultrasound phantom. (b)–(g) B-mode sonograms of ultrasound dummies filled with various components to mimic characteristic findings and artifacts of ultrasound examination of the chest. (b) Liquid filled dummy simulates pleural line and PLE. Note the presence of a needle artifact (long axis); (c) dummy filled with air to mimic reverberation artifacts typically found in PTX, (d) dummy filled with rice grains and starch to mimic partly consolidated hemothorax or fibrinous structures, (e) olive as substitute for soft but solid tissue, (f) jelly babies, and (g) a wooden tiger duck for identification of target objects.

**Figure 2:**
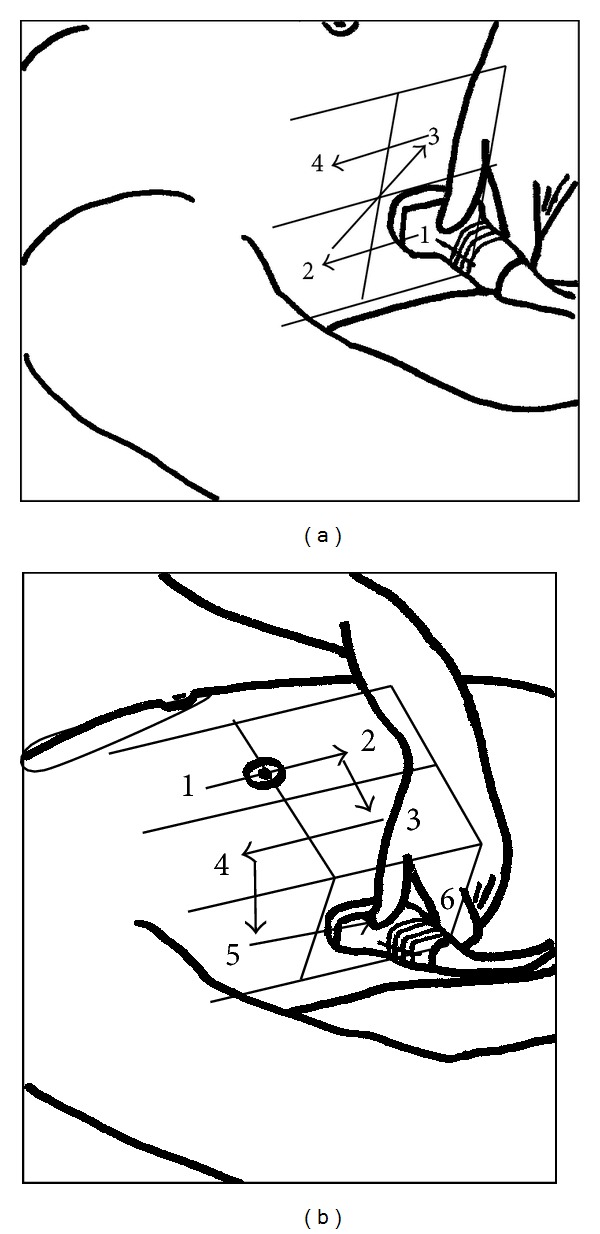
(a)-(b) Standardized training sequence for ultrasound examination of the right hemithorax. Trainees had to examine four views for pleural effusion (a) or six views for pneumothorax (b) by correct positioning of the ultrasound probe.

In order to support the trainees active learning process, we included custom made gel phantoms into the HOTs. Gel was made by commercially available pork skin leafs (Dr. Oetker Nahrungsmittel, Bielefeld, Germany). Phantoms were prepared with 20 grams (equivalent to 12 pieces) of gelling leafs per half a litre of distilled water, carefully heated to 60°C, followed by stirring for 1 min. Next, gels were casted into plastic containers (95 × 15 × 15 cm) and allowed to cool down for two hours at room temperature. While still viscous, water and content filled rubber balloons were carefully submerged in the gel body by avoiding insertion of air bubbles. Next, gels were incubated in a refrigerator overnight until solid. Stiff gel phantoms were used without antimicrobiological additives for 10 days. In HOT-1, one station contained a series of rubber balloons. Each balloon was filled with specific contents empirically chosen to mimic typical phenomena found in chest ultrasound: (a) pure water to visualize transmission and reflexion of ultrasound on interfaces with different acoustic impedance, for example, thorax wall, pleural line, and PLE; (b) air to mimic reverberation artifacts found in PTX; (c) parboiled rice grains and starch to mimic partially consolidated PLE and fibrinous structures; (d) olives as substitute for soft but solid tissues; (e) a stone as substitute for carbon-rich structures such as bones with complete ultrasound absorption and dorsal extinction. Finally, balloons with small jelly babies sized less than 1 cm and a tiger duck made of wood of a size of about 2 cm were included as target structures and to practice recognition of specific objects (Figures 1(a)–1(g)). In HOT-2, one station contained a thoracocentesis set (Pleurofix no. 1, B. Braun, Melsungen, Germany), a 10 mL syringe, and 20 G cannulae (size 0.9 × 0.9 cm). A puncture model was custom made of gel cuboids with immersed rubber balloons (approx. 7 cm in diameter) and prefilled with a clear yellowish liquid, resembling PLE. Each balloon could be used at least two times when reinjected with liquid. Trainees received instruction on how to use thoracocentesis equipment and on techniques of ultrasound-guided puncture, for example, in-line and out-of-plane visualization of the needle (Figure 1(b)). Additionally, trainees were taught a standardized sonographic examination sequence to screen for PLE or PTX (Figures 2(a) and 2(b)). Ultrasound equipment used in this study was SonoSite Titan and MicroMaxx, equipped with a linear array (L38e, frequency range: 10–5 MHz), micro convex array (C15 7.5, 4–2 MHz), or convex array (C60e, 5–2 MHz) probe, kindly provided by SonoSite GmbH, Frankfurt, Germany.

### 2.4. Course Assessment

For scientific evaluation of the THOLUUSE program, trainees had to pass three types of tests: (a) a theoretical test with multiple-choice questions (MCQs) before (precourse) and after (postcourse) completion of the program. Each MCQ test set contained fifteen textural questions and five questions with scanned ultrasound pictures where specific sonoanatomical structures had to be identified. The maximum time to answer each question was 60 seconds. As one of the posttests, (b) a recognition quiz to test the visual perception skill with fifteen video clips (see Appendix Table 1 in Supplementary Material available online at http://dx.doi.org/10.1155/2013/312758), each loop had a duration of 10 seconds followed by a 5-second break to note the results onto a standardized answering form. A self-running DVD was produced with MAGIX 5.5 deluxe (MAGIX AG, Berlin, Germany). The DVD was started once and ran continuously without replay or break until all video clips were shown. The order of the clips, questions, and answers for postcourse testing was changed in a randomized fashion to prevent memory of order or answers. Finally, at the end of HOT-2, there was (c) a practical postcourse examination in which trainees had to demonstrate their skills in correct positioning of the ultrasound probe and to visualize and identify sixteen predefined sonoanatomic items considered particularly relevant to chest ultrasound (see Appendix Table 2). Over 15 min, trainees performance was observed by an instructor and rated on a standardized score sheet. Instructors were not allowed to help or modify the positioning of the probe, and noted results were blinded to the participants. A cut-off level of 60% correctly performed tasks/identified structures was arbitrarily defined to passing before the onset of the study.

### 2.5. Statistical Analysis

Our null hypothesis was that training would not significantly increase trainees skills. A finding was considered significant when error probability was less than 5% and null hypothesis could be rejected. Results are given as box plots, scatter plots, or mean and confidence intervals or standard deviation if not indicated otherwise. For comparison of the training effects of the different test groups, nonparametric Wilcoxon matched-pairs signed rank test (within groups) or Mann Whitney *U*-test (between groups) was applied. Originally, we had scheduled a precourse test for practical skills as well. However, we had to omit precourse testing because of insufficient knowledge and practical skills of most participants before entering the study. Therefore results of the postcourse test were computed against zero.

Test results are shown in two ways: (a) mean result of all trainees passing a test was regarded as general learning effect by examinees and (b) number of trainees who scored positive for a specific question or observation. The latter was interpreted as learning success on a specific item. Statistical analysis and figures were produced with GraphPad Prism software (San Diego, CA, USA).

## 3. Results 

### 3.1. Assessment of Immediate Learning Effects

**Figure 3:**
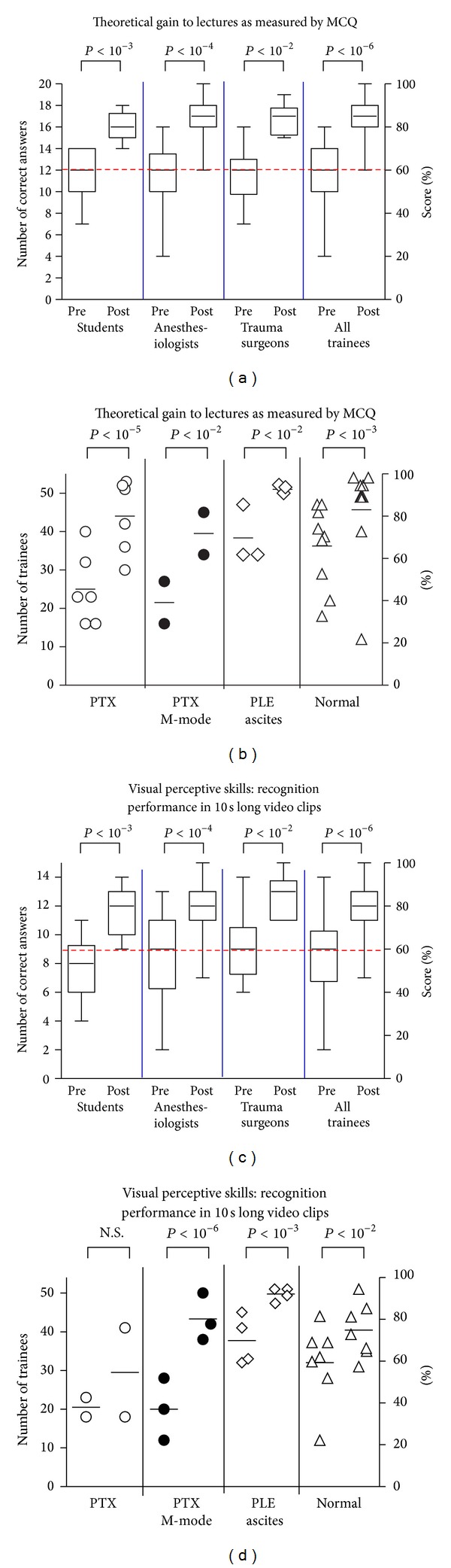
(a) Theoretical learning results of individual trainees and test groups assessed with MCQ in a pre-course (Pre) and post-course (Post) testing. Box plots represent the 25th and 75th percentiles with median. Dashed line indicates an arbitrary defined pass level of 60% (b) Number of trainees who correctly answered MCQ. Each symbol represents an independent question sorted by related categories. (c) Test for visual perceptive skills, where trainees had to identify characteristic physiologic or pathologic ultrasound findings, each shown in a 10 sec video clips. Dashed line indicates an arbitrary defined pass level of 60%. (d) Numbers of trainees who obtained a correct answer during visual perceptive skill test. Each symbol represents a video-clip sorted by related categories.

Medical students (group A), anaesthesiologist residents (group B), and trauma surgeons (group C) mean precourse scores of correct answers were 11.2 (10.5–13.1, 95% CI), 11.5 (9.8–13.1), and 11.8 (9.5–14.0), respectively. After completion of postcourse exams, all groups of trainees achieved comparable increases in their scores: A: 16.1 (15.2–16.1, *P* < 0.001 for comparison to precourse results), B: 17.1 (16.2–17.8, *P* < 0.001), and C: 16.8, (15.6–17.9, *P* < 0.001), respectively (Figure 3(a)). When MCQs were sorted according to the learning target of the course curriculum, the gain of theoretical knowledge in sonoanatomy, physiology, and pathology of the chest, particularly regarding PLE and PTX, was homogeneous in all groups (Figure 3(b)).

### 3.2. Recognition and Interpretation Skills

The ability of the trainees to evaluate a specific physiological or pathological ultrasound finding under pressure of time was tested by recognition and interpretation of video clips. The number of correct answers markedly increased from pre- to postcourse tests in all groups: group A from 7.9 (6.8–9.1) to 11.6 (10.8–12.5, *P* < 0.001 in comparison with precourse testing), in B from 8.5 (6.7–9.2) to 12.3 (10.3–13.2, *P* < 0.001), and in C from 9.1 (7.1–11.2) to 12.6 (11.4–13.9, *P* < 0.01), respectively (Figure 3(c)). When the scores were sorted by pathology, best results were obtained for detection of fluids. Following completion of the course program, most trainees were familiar with basic sonographic signs for detection of PTX as compared to normally inflated lungs by using both B- and M-mode, (Figure 3(d)).

### 3.3. Practical Imaging Performance

**Figure 4:**
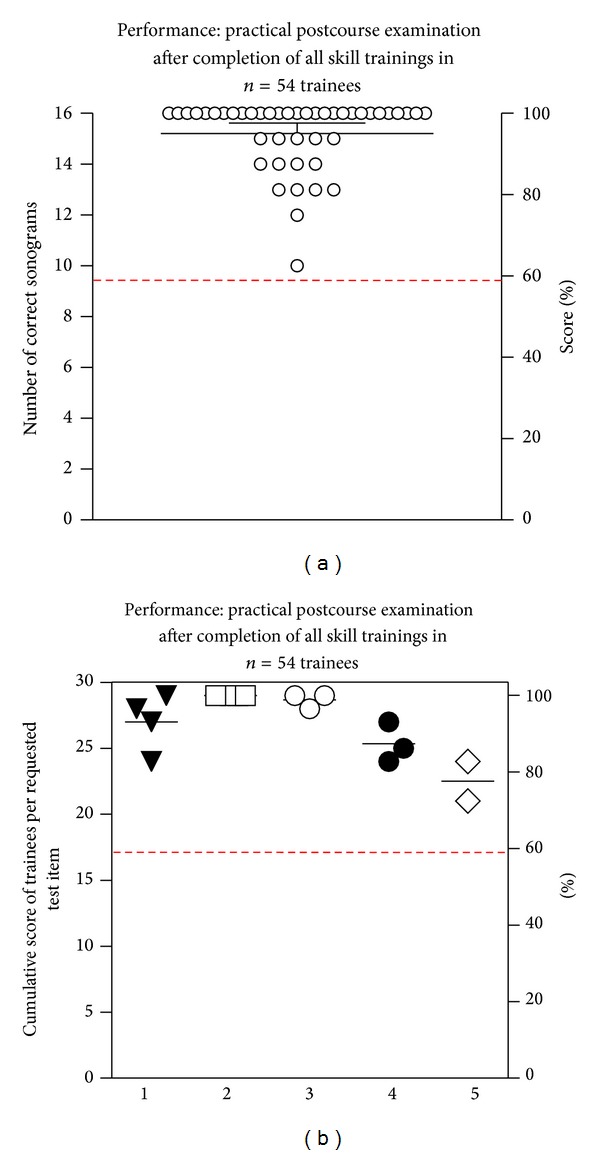
(a)-(b) Practical postcourse examinations after completion of all skill trainings (a) Every circle represents the sum of correct ultrasound examinations of specific sites by individual trainees, as assessed by practical postcourse examination (maximum count: *n* = 16). (b) Each identical symbol represents a distinct learning objective (1: trachea, thyroid, central vessels, and isthmus, 2: chest wall, 3: pleura, lung, 4: diaphragm, solid organs, and 5: detection sequence of PLE or PTX). Data are arranged to visualize the cumulative scores of trainees per tested objective (maximum count: *n* = 54). Dashed line indicates an arbitrary defined pass level of 60% correct answers.

Testing more complex practical capabilities (i.e., steering the probe to a standardized anatomic position, obtaining a clear view, and identifying the sonoanatomical findings/artifacts), a mean of 95% of the postcourse sonograms was conducted in a technically correct fashion (Figure 4(a)).

Subgroup analysis of practical performance skills revealed no statistical significant differences of visualization and interpretation of distinct anatomic sites/learning items (Figure 4(b)).

## 4. Discussion

In the present study, we developed and assessed an OSCE-based training program for THOLUUSE. For more than a decade, FAST has been established as a standard procedure in shock rooms or emergency departments for early diagnosis of cavity bleeding in blunt trauma. Kirkpatrick et al. [1] suggested an extension of FAST (the so-called EFAST) to include sonographic detection of posttraumatic pneumothoraces in the algorithm. However, in the absence of appropriate teaching models and concepts, it remains challenging for an individual examiner to gain sufficient expertise for correct sonographic detection of PTX. Techniques of chest ultrasound, including PTX, have previously been described; however, the effectiveness of those instructions was never assessed and/or validated [1, 16, 22, 23].

It has been questioned if a one-day training course with a brief factual input and few essential exams can teach ultrasound skills that can be of practical relevance for the trainee [24]. It is well known that factual knowledge communicated by noninteractive lectures has only limited impact on gaining and keeping knowledge [25]. With respect to ultrasound examination, it seems rather unlikely that sufficient expertise can be gained from such lectures alone [23]. In the same token, Sisley et al. reported that simply assessing factual knowledge gives an incomplete picture of performance, and they observed low image interpretation skills in the ultrasound evaluation of trauma [23]. Therefore, in this study, we chose an OSCE-based approach that encourages active learning and practical performance. This method is also useful to assess different competencies and can identify/unmask areas with deficiencies that are of practical relevance [23].

Our results show that upon completion of a one-day LUS course participants were able to perform a standardized sonographic examination of the thorax and correctly identify and interpret the most relevant physiological and pathological findings, including PLE and PTX. Of note, we found no significant differences between medical students and postgraduates from different medical professions, and trainees postcourse performances were independent of previous levels of ultrasound experience. Our findings are supported by a project for experimental ultrasound learning, the Advanced Diagnostic Ultrasound in Microgravity (ADUM) [26], launched by the National Aviation and Space Agency (NASA). In this study, nonmedical astronauts were trained by On-board Proficiency Enhancer (OPE) software to send a sonogram to a remote ultrasound expert on earth [27]. The authors demonstrated that sufficient skills for technical conductance and image acquisition of ultrasound can be taught to ultrasound novices in a few hours [26]. Similarly, Bedetti et al. showed that recognition of B-lines is equally reliable whether an experienced or a novice echocardiographer performed the exam [28]. For FAST, it has been shown that a 1-day training course allows trainees to perform a preclinical FAST with a high level of accuracy [19].

A limitation of this study was that postcourse performance of trainees could only be assessed shortly after completion of the program. Previous evaluations of FAST course programs demonstrated that trainees skills decreased as a function of time, particularly when ultrasound techniques were not practiced on a regular basis and further supervised teaching was not available [25]. Therefore, we and others currently are establishing a continuous educational program that combines modules for abdominal, heart, and thorax ultrasound in emergency and critical care medicine [29, 30].

In this respect, the THOLUUSE concept proposed in this study was certified by the German Society of Ultrasound in Medicine (DEGUM) and incorporated substantially into a modular blended learning programme of the German Society of Anesthesiology and Intensive Care Medicine (DGAI) for official training in emergency and critical care medicine [31].

## 5. Conclusion

The results of our study demonstrate that a one-day training program like THOLUUSE significantly improves theoretical and practical skills for sonographic diagnosis of acute thoracic lesions, including PLE and PTX. Postcourse gain of competence was independent of previous ultrasound expertise of the trainees, and we propose to implement THOLUUSE in the training of medical emergencies. THOLUUSE long time impact on the management of patients in emergency and critical care medicine needs further investigation.

## Supplementary Material

Showing the pre- and post-course video simulation test that was used to assess visual perceptive skills and a list of all items the trainees had to identify during the post-course examination.

## Figures and Tables

**Table 1:** Course structure of THOLUUSE.

Program number and time limit	Lecture, case presentation, or HOT^a^	Content and key messages
1 (15 min)	Reasons for thorax, trachea, and lung ultrasonography in emergency and critical care medicine	Introduction, context, advantages, and disadvantages of chest ultrasound in emergency and critical care medicine

2 (15 min)	Sonoanatomy of the thorax, trachea, and lung	Brief physics of US, probes, chest wall and organ anatomy, general remarks on B-mode of structures, basic windows and artifacts, and impact on views of artificial ventilation

3 + 4 (10 min + 5 each)	Two related clinical case presentations from the emergency department (incl. 10 min discussion)	Authentic clinical example, well prepared with original US sequences, relevant PLE, and rib/sternal fracture

5 (20 + 10 min)	US of PLE: phenomena and artifacts (incl. 10 min discussion)	Repetition of basic windows and artifacts, B-Mode, and appearance and differential diagnosis of hypoechogenic findings (pulmonary embolism, fluids, chest hematoma, and lung contusion)

(90 min)	HOT-1	Practical training with instructors and models

6 (15 min)	US of trachea, cartilages, and cricoids: sonogram and artifacts	Air artifacts, reverberation, and procedural ultrasound use within percutaneous dilatational tracheotomy

7 (20 + 10 min)	US of pneumothorax: sonogram and artifacts (incl. 10 min discussion)	Views of air artifacts, reverberation, loss of pleural sliding and comet tails, lung pulse, and understanding M-mode sonograms

8 (15 min)	Standardized sequence of lung ultrasonography	How to quickly examine a patient with suspected PLE or PTX. Algorithm training with practical relevance for time sensitive scenarios.

9 + 10 (10 min + 5 each)	Two related clinical case presentations from intensive care medicine (incl. 10 min discussion)	Physiology of lung US in intensive care medicine, postprocedural (insertion of central line), postcardiotomy PTX despite tube drain

(90 min)	HOT-2	Practical training with instructors and models

^a^Lectures and cases all included a brief discussion; all major artifacts were taught in a clinical context. Lectures were followed by hands-on trainings (HOT).

PLE: pleural effusion; PTX: pneumothorax; US: ultrasound examination.

**Table 2:** Stations and learning targets of hands-on training (HOT) stations.

Station no. HOT-1/HOT-2	Training: station topic	Model/patient and position	Content/learning issue	Scan mode B/M
1 (HOT-1)	Thorax	Model, sitting	Thorax, ribs, bone, cartilage, and sternum	B
2 (HOT-1)	Pleura	Model, supine	Lung sliding, B-lines	B
3 (HOT-1)	Differential diagnosis	Model, supine	Lung, lateral and posterior axillary lines, liver, spleen	B
4 (HOT-1)	Ultrasound phantom	—	Training on gel-embedded artifacts in rubber balloon US-phantoms	B
5 (HOT-1/2)	Trachea	Model, supine	Trachea, central and subcutaneous vessels, cricoid cartilage, and thyroid gland	B
6 (HOT-1/2)	“Virtual” station	Laptop, screen	12 pictures divided in HOT-1 and HOT-2 with or without pathologies, explained by instructor	—
7 (HOT-1/2)	US sequence	Dummy and model, supine	Training of sequence on manikins (HOT-1) and models (HOT-2)	B
8 (HOT-2)	Advanced lung- and pleural US	Model, supine	Apnea, “lung pulse” and “seashore” sign	B/M
9 (HOT-2)	Advanced lung US	Patient sitting/supine	Training with patient and pathology (atelectasis, PLE, or PTX)	B/M
10 (HOT-2)	US sequence	Patient, supine	Training of algorithm and sequence with patient and pathology (atelectasis, PLE, or PTX)	B/M
11 (HOT-2)	Puncture phantom	—	Pleural effusion, puncture gel phantom	B

**Table 3:** Learning targets of the “virtual station” within the hands-on training (HOT).

Picture number	Related topic	Mode (B/M)	Details to recognize	Difficulty level
1	Normal and edema	B	A-line, reverberation artifacts, multiple B-lines	2
2	Normal	B	Peritoneum, kidney, and bony rib artifact with posterior acoustic shadowing	1
3	Fluid differential diagnosis	B	Four B-mode views of fluids: subdiaphragmatic fluid and liver, ascites, spleen, and diaphragm, PLE, lobe atelectasis, diaphragm and liver, and ascites and small bowel	1
4	PLE	B	Spleen, fluid, and compression atelectasis	1
5	PLE/ascites	B	Small amounts of PLE, diaphragm, and ascites	2
6	Acoustic shadowing, anatomy and, stone	B	Liver and hyperechogenic diaphragm, gall bladder and stone with posterior acoustic shadowing	1
7	PLE, M-mode appearance	B and M	Small PLE, multiple comet tails, A-line, and separated visceral pleura	2
8	PLE	B	Large amount of PLE, good view of diaphragm and spleen	1
9	Peripheral pulmonary embolism	B	Visible triangular break in visceral pleural line due to peripheral pulmonary embolism, lung tissue	2
10	Lung pulse, normal M-mode	B and M	Reverberation artifacts of pleural line, lung pulse, sonoanatomical finding of “seashore” sign in the M-mode	2
11	Stratosphere sign	B and M	Multiple reverberation artifacts, pleural line	2
12	Lung point	B and M	Breakup of pleural line (change point between seashore/stratosphere sign)	2

PLE: pleural effusion; PTX: pneumothorax; US: ultrasound examination.

## Abbreviations

FAST: Focused abdominal sonography for trauma
LUS: Lung ultrasound
HOT: Hands-on training
MCQ: Multiple choice question
OSCE: Objective structured clinical examination
PTX: Pneumothorax
PLE: Pleural effusion
THOLUUSE: Thorax, trachea, and lung ultrasonography in emergency and critical care medicine.
