# Supplementary material for: Thorax, Trachea, and Lung Ultrasonography in Emergency and Critical Care Medicine: Assessment of an Objective Structured Training Concept
Source: Emerg Med Int. 2013 Nov 27;2013:312758. doi: 10.1155/2013/312758 (PMC3863481; doi:10.1155/2013/312758)
Supplement: Supplementary file 1 — Showing the pre- and post-course video simulation test that was used to assess visual perceptive skills and a list of all items the trainees had to identify during the post-course examination. [file 312758.f1.docx]

**Appendix**

**Table 1:** Pre- and post-course video clip simulation test to assess visual perceptive skills.

| **Movie Clip^a^** | **Related Topic** | **Mode (B/M)** | **Details to recognize** | **Difficulty level** |
| --- | --- | --- | --- | --- |
| **1** | PTX | B | Lung-point, loss of Lung-sliding | 3 |
| **2** | PLE | B | Fluid and lobular atelectasis | 1 |
| **3** | NOR | M | Pleura, reverberation and seashore sign | 2 |
| **4** | PTX | B | No lung sliding, multiple reverberations | 2 |
| **5** | ASC | B | Abdominal fluid, diaphragm, spleen | 1 |
| **6** | NOR | B | Lung sliding | 1 |
| **7** | ASC | B | Abdominal fluid, liver | 1 |
| **8** | PTX | B and M | Combined information with normal and PTX, lung point in M-Mode | 3 |
| **9** | PLE | B | Fluid, diaphragm, lobe atelectasis | 1 |
| **10** | NOR | B | Zoom of cutis, subcutis, muscle and pleural gliding, moving comet tail | 1 |
| **11** | PTX | M | No lung sliding, multiple reverberation artifacts | 3 |
| **12** | NOR | B | Moving B-line, lung sliding | 1 |
| **13** | PTX | B | Absence of B-line or lung sliding | 2 |
| **14** | PLE | B | Small amount of fluid, compression atelectasis | 1 |
| **15** | PLE | B | Large amount of fluid with compression atelectasis | 1 |

NOR, normal; PLE, pleural effusion; PTX, pneumothorax, ASC, ascites

^a^A self-starting DVD presented video clips with varying levels of difficulty. The duration of each clip was 10 seconds. In a 5 second pause without showing the clips or replay, trainees had to check an answer on a score-sheet.

**Appendix**

**Table 2:** List of test items, trainees had to identify within the post-course examination.

| **Test item**  **Number** | **Exam Topic** | **Mode (B/M) and axis** | **Content to show to the instructor in the sonogram^a^** | **Difficul-ty level** |
| --- | --- | --- | --- | --- |
| **1** | Trachea | B, SAX and LAX | Identify trachea (SAX), show tracheal cartilages (LAX) | 1 |
| **2** | Thyroid gland | B, SAX | Show tissue and organ in a cranio-caudal sweep | 1 |
| **3** | Isthmus of Thyroid gland | B, SAX | Show details in relation to trachea | 2 |
| **4** | Central collar vessels | B, SAX | Identify and show topology to trachea | 1 |
| **5** | Cutis | B | Identify and demonstrate | 1 |
| **6** | Subcutis | B | Show difference of fatty tissue and muscle | 1 |
| **7** | B-Line | B | Find and show movement | 2 |
| **8** | Ribs | B, SAX | Show bony or cartilaginous ribs | 1 |
| **9** | Ribs | B | Show posterior acoustic shadowing | 1 |
| **10** | Pleura | B/M | Identify and show pleura and reverberations in B- and M-Mode | 1 |
| **11** | Lung sliding | B/M | Identify and show lung sliding in B- and M-Mode | 2 |
| **12** | Sequence for detecting PTX | B | Show how to do (6 defined positions for obtaining scans) | 2 |
| **13** | Liver | B, cranio-caudal | Show organ and sweep organ distance | 1 |
| **14** | Spleen | B, cranio-caudal | Show organ and sweep organ distance | 1 |
| **15** | Diaphragm | B | Identify and show in relation to liver, spleen and lung | 1 |
| **16** | Sequence for detecting PLE | B | Show how to do (4 defined positions for obtaining scans) | 2 |

PLE, pleural effusion; PTX, pneumothorax; SAX, short axis; LAX, long axis

^a^ Each item was subject to acceptance or rejection by the instructor and evaluation was blinded to the trainee. Whenever a trainee showed an item correctly, one single point was awarded each to receive a cumulative score. Short axis (SAX); long axis (LAX)
